# Treatment of palmoplantar keratoderma in a patient with ichthyosis with topical tapinarof

**DOI:** 10.1016/j.jdcr.2024.01.017

**Published:** 2024-01-27

**Authors:** Sarah N. Robinson, Julie S. Kranseler, David Rosmarin

**Affiliations:** a Department of Dermatology, Tufts Medical Center, Tufts University School of Medicine, Boston, Massachusetts; b Department of Dermatology, Indiana University School of Medicine, Indianapolis, Indiana

**Keywords:** AhR, aryl hydrocarbon receptor, ichthyosis, Nrf2, palmoplantar keratoderma, tapinarof cream

## Introduction

Palmoplantar keratodermas (PPKs) are a heterogeneous group of hereditary or acquired disorders characterized by abnormal thickening of the skin of the palms and soles, often with painful fissures that may impact ambulation and thus quality of life. Treatments are typically aimed at providing symptomatic relief with varying effectiveness and possible side effects, including emollients, keratolytics, retinoids, and corticosteroids.^1^^,^^2^ Tapinarof is a topical aryl hydrocarbon receptor and NF-E2–related factor 2 (Nrf2) modulating agent that was recently approved for the treatment of plaque psoriasis. In this article, we describe a patient with congenital ichthyosis who had notable clinical and symptomatic improvement in PPK of the soles while treating with tapinarof.

## Case Report

**Fig1:**
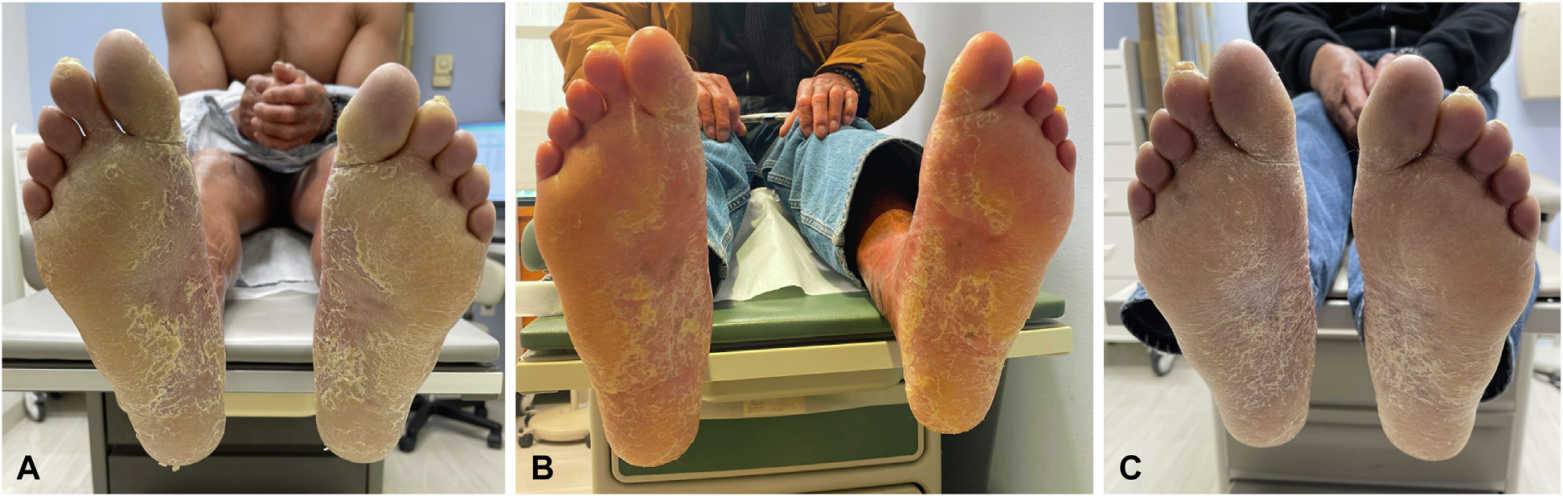
**A,** Prior to treatment with topical tapinarof. **B,** After 4 weeks of treatment with topical tapinarof. **C,** After 10 weeks of treatment with topical tapinarof.

A 62-year-old man presented with a history of ichthyosis since childhood, initially erythrodermic at birth then subsequently with diffuse scaling and itch on the trunk and extremities, as well as progressive PPK with painful fissures on palms and soles. On examination he had large plate-like scale most pronounced on his extremities and hyperkeratotic yellowish plaques on plantar feet and palms consistent with congenital ichthyosis with PPK (Fig 1, *A*). A saliva sample was sent for genome analysis. Whole exome sequencing was performed, which did not reveal any mutations in currently known disease-causative genes for ichthyosis and PPK. His treatment regimen included acitretin 25 mg daily and ammonium lactate lotion twice daily. He had previously tried numerous other treatments for his scale, eczematous rash, and pruritus including urea (10%-40%), salicylic acid, tacrolimus, crisaborole, high and ultrahigh potency topical steroids, dupilumab, abrocitinib, and ixekizumab, all with minimal improvement. He was then started on treatment for his PPK with tapinarof 1% cream, applying once daily to affected areas, focusing on the soles. After 4 weeks of treatment, patient reported improvement in the thickness of his soles (Fig 1, *B*). After 10 weeks of treatment, he noticed further improvement of his soles, which on clinical examination were noted to be thinner and less hyperkeratotic with resolution of painful fissures (Fig 1, *C*). The patient was stable on acitretin 25 mg daily and had no changes to other drugs during the course of treatment. Of note, treatment was during the cold winter months, typically the most problematic time of year for his skin. The patient denied any side effects from treatment.

## Discussion

This case describes improvement in PPK of the soles in a patient with ichthyosis using topical tapinarof. Tapinarof has a novel mechanism of action as an aryl hydrocarbon receptor and Nrf2 agonist that is thought to be effective for treatment of psoriasis based on its ability to downregulate interleukin 17. As part of its mode of action it also decreases other aspects of inflammation and enhances skin barrier through impact on filaggrin and loricrin in keratinocytes.^3^ In Krt16-null mice, Nrf2 is hypoactive and activation of Nrf2 prevents PPK-like lesions.^4^ We hypothesize that tapinarof’s ability to agonize Nrf2, reduce oxidative stress, and increase skin barrier function is how it can improve PPK. Our patient noted gradual improvement with apparent decrease in thickness of his soles as well as symptomatic improvement within 10 weeks of treatment with tapinarof. Importantly, he did not report any side effects during treatment. Furthermore, he may have been at lower risk of experiencing folliculitis, the most common side effect of topical tapinarof, as he was only treating non–hair-bearing areas.^5^ Given the limited number and efficacy of therapies currently available for patients with ichthyosis and PPK, along with the need for lifelong treatment, topical tapinarof may be a viable alternative treatment option for these patients. Additional studies are needed to further elucidate the mechanism of action of tapinarof for treatment of PPK and to help identify which patients would benefit most from treatment.

## Conflicts of interest

Dr Rosmarin has received honoraria as a consultant for AbbVie, Abcuro, AltruBio, Arena, Boehringer Ingelheim, Bristol Myers Squibb, Celgene, Concert, CSL Behring, Dermavant, Dermira, Incyte, Janssen, Kyowa Kirin, Lilly, Novartis, Pfizer, Regeneron, Recludix, Revolo Biotherapeutics, Sanofi, Sun Pharmaceuticals, UCB, and Viela Bio; has received research support from AbbVie, Amgen, Bristol Myers Squibb, Celgene, Dermira, Galderma, Incyte, Janssen, Lilly, Merck, Novartis, Pfizer, and Regeneron Pharmaceuticals Inc; and has served as a paid speaker for AbbVie, Amgen, Bristol Myers Squibb, Celgene, Dermavant, Incyte, Janssen, Lilly, Novartis, Pfizer, Regeneron Pharmaceuticals Inc, and Sanofi. Drs Robinson and Kranseler have no conflicts of interest to declare.
